# High-Density Electroencephalography-Informed Multiband Functional Magnetic Resonance Imaging Reveals Rhythm-Specific Activations Within the Trigeminal Nociceptive Network

**DOI:** 10.3389/fnins.2022.802239

**Published:** 2022-05-16

**Authors:** Hauke Basedau, Kuan-Po Peng, Arne May, Jan Mehnert

**Affiliations:** Department of Systems Neuroscience, University Medical Center Hamburg-Eppendorf, Hamburg, Germany

**Keywords:** simultaneous EEG-fMRI, trial-to-trial variance, beta time-series, correlation, validation, brain rhythms

## Abstract

The interest in exploring trigeminal pain processing has grown in recent years, mainly due to various pathologies (such as migraine) related to this system. However, research efforts have mainly focused on understanding molecular mechanisms or studying pathological states. On the contrary, non-invasive imaging studies are limited by either spatial or temporal resolution depending on the modality used. This can be overcome by using multimodal imaging techniques such as simultaneous functional magnetic resonance imaging (fMRI) and electroencephalography (EEG). Although this technique has already been applied to neuroscientific research areas and consequently gained insights into diverse sensory systems and pathologies, only a few studies have applied EEG-fMRI in the field of pain processing and none in the trigeminal system. Focusing on trigeminal nociception, we used a trigeminal pain paradigm, which has been well-studied in either modality. For validation, we first acquired stand-alone measures with each imaging modality before fusing them in a simultaneous session. Furthermore, we introduced a new, yet simple, non-parametric correlation technique, which exploits trial-to-trial variance of both measurement techniques with Spearman’s correlations, to consolidate the results gained by the two modalities. This new technique does not presume a linear relationship and needs a few repetitions per subject. We also showed cross-validation by analyzing visual stimulations. Using these techniques, we showed that EEG power changes in the theta-band induced by trigeminal pain correlate with fMRI activation within the brainstem, whereas those of gamma-band oscillations correlate with BOLD signals in higher cortical areas.

## Introduction

The human trigeminal nociceptive system is the origin of numerous pathologies such as headaches and facial pain syndromes (Stankewitz et al., 2010; Schulte et al., 2016, 2017; Goadsby et al., 2017; Mehnert et al., 2017). Most studies on humans are limited by either spatial or temporal resolution, depending on the modality used. While electroencephalography (EEG) provides the high temporal resolution needed for casual interference such as coupling measures, it lacks spatial resolution. In contrast, functional magnetic resonance imaging (fMRI) can provide a spatial resolution of up to 1 mm^3^, yet it suffers from poor temporal resolution. The idea of combining both non-invasive techniques through simultaneous measurements thus has unique potential (Mulert and Lemieux, 2010) because it may overcome the spatial constrain of the EEG and the temporal limitation of fMRI. However, it is methodologically demanding and although neuroimaging of trigeminal nociception has made substantial progress in understanding trigeminal processing and related pathophysiology (May, 2013), simultaneous EEG-fMRI has not yet been established for trigeminal nociception. Here, we explored simultaneous EEG-fMRI as an intriguing tool to enhance insights into the human trigeminal nervous system.

Simultaneous EEG-fMRI has not yet been established to research the human trigeminal pain system. Nevertheless, a few studies using EEG-fMRI offer insights into the pain processing of other parts of the body (Iannetti and Mouraux, 2010). These studies mostly used heat delivered either by thermode (Roberts et al., 2008; Mayhew et al., 2013) or laser (Iannetti et al., 2005; Mobascher et al., 2009a,b; Brinkmeyer et al., 2010) on the hand, arm, or leg. One study applied painful electrical stimulation (Christmann et al., 2007), but the stimulations mentioned in these studies are not easily transferable to investigate the trigeminal nociception and furthermore exploit event-related potentials (ERP) rather than event-related synchronization (ERS) and desynchronization (ERD) of individual frequency bands, which are more robust to shifts of the stimulation onset in the millisecond range.

To investigate the spatiotemporal mechanisms of physiological trigeminal pain processing non-invasively in humans, we used a standardized and well-published experimental study design, eliciting trigeminal pain by applying gaseous ammonia into the nostril. Further conditions include visual stimuli as well as rose odor and simple air puffs as control conditions (Stankewitz et al., 2010; Schulte et al., 2016). We acquired high-density EEG using 64 channels (Scarff et al., 2004) and simultaneously fMRI with a brainstem optimized protocol (Schulte et al., 2016) that was extended through multiband acquisition techniques (Uji et al., 2018) to cover also all cortical areas of the brain. Our data fusion of both measurement modalities (EEG and fMRI) reveals new insights into the spatiotemporal dynamics of the trigeminal nociceptive system (May et al., 2020).

The aim of the study was twofold. First, we aimed to verify a novel analytical routine for fusing fMRI and EEG data. To this end, we used non-parametric Spearman’s correlations between single-trial EEG power changes and single-trial blood oxygen level-dependent (BOLD) changes from the fMRI. This validation was used in the visual condition first, as a correlation between the induced steady-state evoked potential and the occipital regions of the brain is rather robust. In a second step, we aimed to gain deeper insight into trigeminal nociception by using the aforementioned analytical routine to correlate evoked EEG features with the fMRI during painful trigeminal input.

For the visual control condition, we aimed to replicate an (early) event-related potential (ERP), decreased alpha event-related synchronization (ERS), and, most prominently, a steady-state evoked potential (SSEP) and its higher harmonics (Mehnert et al., 2019) in the time-frequency representation of the central occipital electrode (Oz) of the EEG. The SSEP should correlate with BOLD changes in occipital regions in the fMRI.

The painful stimulation is estimated to reproduce the previously presented results in EEG. This refers to an ERP representation in the theta-/delta-band (Ploner et al., 2006; Huart et al., 2012; Taesler and Rose, 2016; Mehnert et al., 2019), a decrease in the alpha-band (Mehnert et al., 2019), and an increase in gamma ERS (Bader, 2019), all at the central-parietal electrode Pz.

We further hypothesized that correlations between power changes of the theta frequency and hemodynamics of the fMRI are present in areas in the brainstem pertinent to the trigeminal nociceptive system, including the spinal trigeminal nucleus (STN), the rostral ventromedial medulla (RVM), and eventually the periaqueductal gray (Stankewitz et al., 2010). We further expected gamma ERS to correlate with cortical areas of the pain matrix (Zhang et al., 2012).

## Materials and Methods

### Subjects

In total, 35 healthy volunteers (18 women, age: 28.03 ± 3.94 years) participated in a standardized experiment on trigeminal pain processing. The study was approved by the Local Ethics Committee in Hamburg, Germany (PV 4896) and was conducted in accordance with the Declaration of Helsinki. We obtained written informed consent before the initiation of the first study session. The volunteers underwent a two-session pilot study consisting of (i) acquisition of EEG solely and (ii) acquisition of fMRI solely. Of these 35 participants, 18 volunteers were recruited for a final third session of (iii) simultaneous acquisition of EEG-fMRI. One was excluded due to inadequate data quality and prominent fMRI-artifact residuals in the EEG. The criteria for insufficient data quality are described in the section “Preprocessing of Electroencephalography Data.” Therefore, the final group of combined EEG-fMRI consisted of 17 (9 women, age: 28.29 ± 3.51 years) datasets.

### Experimental Design

**FIGURE 1:**
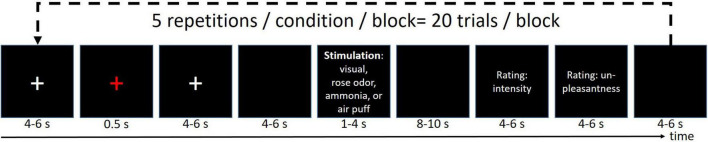
Experimental design. Each run was repeated three times resulting in 15 stimuli/condition/participant.

The stimulus design is well-established and has been published multiple times elsewhere (Stankewitz et al., 2010; Schulte et al., 2016; Mehnert et al., 2017, 2018, 2019; Mehnert and May, 2019). In short, the volunteers received four different stimuli (ammonia, rose scent, or air into the left nostril, and a repetitive visual stimulation at 8 Hz) with an interstimulus interval of 46 ± 9 s, where the ammonia elicits a short-lasting, stinging, or stabbing painful sensations (Hummel and Kobal, 1992; Stankewitz et al., 2010). Refer to Figure 1 for an overview of the experimental timeline. For all experimental sessions (EEG alone, fMRI alone, EEG-fMRI), the experiment was divided into three blocks, in which each condition was randomly presented five times, corresponding to 15 presentations of each condition for each participant in total. The subject rated the intensity and unpleasantness after each stimulus using a visual analog scale ranging from 0 to 100 for the intensity rating, where 0 means no pain at all while 100 refers to the worst imaginable pain. The unpleasantness was rated between −50 (extremely pleasant) and 50 (extremely unpleasant).

### Stand-Alone Acquisition of Electroencephalography (First Session)

During the first session of the experiment, we acquired fast (5,000 Hz), high-density EEG (BrainAmp MR plus, Brain Products, Munich, Germany) with 59 channels, as well as echocardiogram (ECG) and 4 electrooculogram (EOG) channels, in a shielded EEG recording chamber. We used a custom-built photoionization detector (PID) (Bentekk, Hamburg, Germany) to track the gaseous stimulation boli of the ammonia with high-temporal resolution (100 Hz) necessary to identify the precise stimulus onset for the EEG analysis and to control the concentration and amount of ammonia given at a single-trial level.

### Stand-Alone Acquisition of Functional Magnetic Resonance Imaging (Second Session)

During a second appointment, high-resolution structural images using an MPRAGE sequence (voxel size 1 mm × 1 mm × 1 mm) were initially recorded, along with field maps (74 slices, 3 mm × 3 mm × 2 mm resolution, FOV 222 mm, TR 0.814 s) for the correction of inhomogeneities in the magnetic field. The participants subsequently underwent high resolution (1.25 mm × 1.25 mm × 2 mm), multiband BOLD fMRI [echo-planar imaging, TR 3.173 s, TE 35 ms, 74 slices, 2 slices at a time (i.e., multiband), FOV 225 mm] covering the brainstem from foramen magnum up to all cortical areas using a 3T MR scanner (PRISMA, Siemens, Erlangen, Germany).

### Acquisition of Simultaneous Electroencephalography and Functional Magnetic Resonance Imaging (Third Session)

For those participating in the third session, simultaneous EEG and fMRI were recorded using the parameters already described for the EEG and fMRI stand-alone sessions. Additionally, a second ECG and pulse and breathing were acquired with a supplementary device (Expression MR-Monitor, PHILIPS Corporation, Massachusetts, United States) to correct for cardiovascular artifacts. The timing of the EEG and the MR scanner was synchronized using a SyncBox (Brain Products, Munich, Germany), and triggers occurring at each radiofrequency pulse (RF-pulse) were passed from the MR to the EEG. All settings followed the protocol provided by the EEG manufacturer (sampling rate = 5,000 Hz; resolution: 0.5 μV; low cutoff = 10 s; high cutoff = 250 Hz; series resistor values = 10 kΩ) (Brain Products, Munich, Germany). During the acquisition, the helium compressor was turned off to avoid vibrations and, therefore, electrical noise for optimizing data quality. An overview of the complete setup is sketched in Supplementary Figure 1.

### Preprocessing of Electroencephalography Data

Electroencephalography data were re-referenced to the average, cut into epochs between −500 and 3,000 ms time-locked to stimulus onset, and high-pass filtered at 0.5 Hz using the FieldTrip toolbox (Oostenveld et al., 2011). Power line artifacts were reduced by a notch filter at 50 Hz. Eye movement and blinking artifacts were automatically eliminated by regressing the difference in the signal between the two vertical as well as the two horizontal EOG channels using the procedure described by Parra et al. (2005). Thereafter, all trials passed the automated muscle detection routine of FieldTrip (version 22-02-2017)^1^ using the *ft_artifact_muscle* routine with default parameters (bandpass-filter: Butterworth at 110–140 Hz, filter order 8, Hilbert transform, and a boxcar of 0.2) and an overall z-score higher than 5 using the *ft_artifact_zvalue* routine, which thresholds the *z*-transformed value of the preprocessed raw data at a z-score of 5. Furthermore, for the investigation of gamma oscillations, it was necessary to filter residual saccades. For this purpose, an algorithm developed by Hassler et al. (2011) was used, which detects transient saccades by an automated decomposition of saccades and removes them from the remaining data by interpolation. All identified artifact-loaded trials were completely excluded from further analysis, leaving 96.38% of the trials (90.7% in ammonia condition, 98.7% in rose, 96.6% in checker, and 99.6% in air condition) for the analysis.

Time-frequency transformation of the individual trials was calculated using the multitaper method (Thomson, 1982; Mitra and Pesaran, 1999; Litvak et al., 2011) for frequencies of 2–100 Hz with frequency steps of 1 Hz and frequency resolution of 1–10 Hz, depending on the frequency under observation {higher resolution for higher frequencies created with MATLAB’s *linspace* [1, 10 length (frequencies)] routine} using the implementation of the SPM12 toolbox^2^. Temporal resolution was set to 800 ms and the temporal steps to 50 ms. The resulting time-frequency spectra were—on a single-trial level—recalculated as relative changes to baseline (defined as 500–0 ms before stimulus onset) by division, logarithmically transformed, and then averaged within the individuals showing the induced responses (David et al., 2006) following the robust averaging protocol within SPM12. Then, the individual averages were cropped to a temporal window from 0 to 2,500 ms regarding stimulus onset.

For comparability reasons, the same approach was chosen for the simultaneous data. Given the synchronization between EEG and fMRI, as stated before and markers for each RF pulse, gradient artifact were corrected using the software provided by the EEG manufacturer (Brain Vision Analyzer 2, Brain Products, Munich, Germany) using 111 gradient template averages (three times the 37 slices) before any other preprocessing step. This algorithm in principle uses an average of several EEG periods as a template for a scanner artifact and subtracts this curve from the data as described in the study by Allen et al. (2000). Cardiobalistic artifacts were also corrected with the aforementioned software using the pulse signal acquired by the Expression ^®^ monitor for the detection of heartbeats. Again, this algorithm uses averages of several pulses used as templates to correct the EEG signals (Allen et al., 1998). In addition, we used a band-stop filter to denoise the remaining artifacts of the fMRI in the frequency range between 11.17 and 12.16 Hz (0.5 Hz around the repetition frequency of the RF pulses, i.e., number of slices/TR). The artifact correction of the preprocessing routine for the simultaneous with fMRI acquired EEG data left 94.0% of the trials (88.2% in ammonia condition, 96.1% in rose, 95.3% in checker, and 96.5% in air condition) for the analysis.

For the visual condition, we extracted trial-wise averages at the stimulation frequency of 8 Hz in the temporal window between 100 and 2,000 ms at the central occipital electrode, where the SSEP is expected. This was previously reported to show a significant increase for the current experimental design (Mehnert et al., 2019). For the nociceptive condition, we extracted trial-wise averages for four time-frequency windows at the central parietal electrode (Pz), which is a representation of the ERP in the theta-/delta-band (Ploner et al., 2006; Huart et al., 2012; Taesler and Rose, 2016; Mehnert et al., 2019), a decrease in the alpha-band (Mehnert et al., 2019) signifying a rise in attention, as well as an increase in gamma ERS (Bader, 2019) as stated in the section “Introduction.” The details of the time-frequency windows are presented in Table 1 and have previously been reported to contain significant changes in response to trigeminal nociception (Grosser et al., 2000; Bader, 2019; Mehnert et al., 2019). Averages of the time-frequency windows used for the nociceptive condition were also tested for the control condition (air puffs). The significance of these derived features is tested by a two-sided *t*-test against 0 with an alpha level of 0.05 in the stand-alone EEG session and for the EEG in the simultaneous EEG-fMRI session.

**TABLE 1:** Time-frequency windows of interest derived from the study by Bader (2019) and Mehnert et al. (2019).

Electrode	Frequency	Time	*t*-value for EEG-standalone/EEG in EEG-fMRI (df = 34/df = 16)	*p* for EEG-standalone/EEG in EEG-fMRI
**Repetitive visual stimulation**				
Oz	8 Hz (flicker, SSVEP)	100–2000 ms	6.16/4.34	<0.0001/<0.0001
**Trigeminal nociception**				
Pz	3–6 Hz (theta/delta)	350–1150 ms	3.72/1.53	0.0007/0.0028
Pz	9–10 (alpha)	1250–2000 ms	−6.10/n.s.	<0.0001/0.2551
Pz	33–43 Hz (low gamma)	100–2000 ms	4.05/2.44	0.0003/0.0266
Pz	57–100 Hz (high gamma)	300–2000 ms	6.13/3.43	<0.0001/0.0034

### Preprocessing of Functional Magnetic Resonance Imaging Data

All fMRI images in the second and third sessions were first denoised using the spatially adaptive non-local mean algorithm (Manjón et al., 2010) implemented in the CAT12 extension of SPM12. Field maps were preprocessed and used for realignment and unwarping of the fMRI data. The data were further corrected for slice time, taking the multiband acquisition into account. Subsequently, functional images were co-registered to the anatomical images; the latter was then used to normalize all data to MNI space with a non-linear approach and smoothed with a 4 mm^3^ isotropic Gaussian kernel (Schulte et al., 2016) for the trigeminal nociception but 6 mm for the repetitive visual stimulation. Data from the three acquired runs were combined into a single general linear model (GLM). In the GLM, we modeled the four conditions (ammonia, visual, rose, and air puffs) as well as the evaluations (ratings) in separate regressors. The GLM further included the run-wise movement parameters calculated in the realignment as regressors of no interest. In a similar fashion, 18–20 regressors per session inferred the cardiac and breathing characteristics of each image using the approach provided by Deckers et al. (2006). Group level statistics were calculated using SPM12: The main effects of the repetitive visual stimulation and the trigeminal nociception (beta images) were statistically tested with a *t*-test at a voxel-wise FWE-corrected threshold of *p* < 0.05 with a minimal cluster extent of 30 voxels in the stand-alone fMRI session. This high statistical threshold was used to ensure the reproductive capacity of the experimental design used.

### Simultaneous Electroencephalography-Functional Magnetic Resonance Imaging Data Fusion

To perform our analytical approach on correlations of trial-to-trial variability in the spirit of Iannetti and Mouraux (2010), we calculated one GLM where each painful, each visual, and each air puff trial were individually modeled with a HRF and included as regressors of interest in a trial-by-trial GLM (Rissman et al., 2004; Abdulrahman and Henson, 2016), while rose scent stimulation was included as a single, condition-wise regressor. As for the stand-alone analysis of the fMRI, further regressors were implemented to account for movement and breathing as well as pulse-related artifacts. The resulting so-called beta time-series (Abdulrahman and Henson, 2016) of each participant was then normalized to MNI space using the SPM12 standard procedure (Ashburner and Friston, 2005) with an isotropic voxel size of 2 mm^3^ using the segmentation of the participants’ structural image. The images were then z-transformed within each subject and concatenated across subjects for visual stimulation, trigeminal nociception, and the control condition (air puffs), respectively.

**FIGURE 2:**
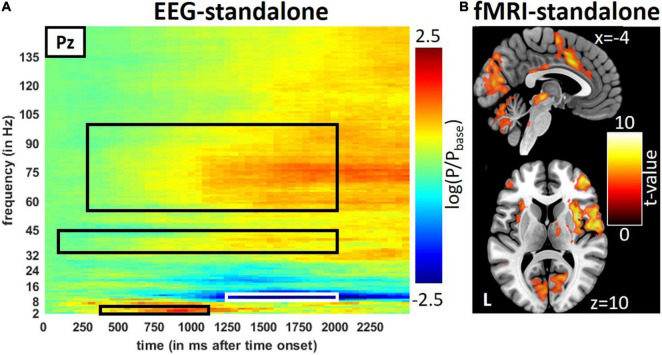
Result from the stand-alone measurements during trigeminal nociception in EEG and fMRI. **(A)** Averages of stimulus induce power changes in time-frequency bands of trigeminal nociception in the EEG and **(B)** activity seen by the fMRI. Results of the fMRI are presented at a visualization threshold of *p* < 0.001 (uncorrected). Time-frequency windows of interest are framed in black or white.

Trial-wise averages of EEG data from one time-frequency window for the visual condition were extracted [the flicker frequency of 8 Hz known to produce an SSVEP (Norcia et al., 2015)] at the central occipital electrode Oz and averages within four time-frequency windows for the trigeminal nociception and the control condition (air puffs) at the central-parietal electrode Pz. The time-frequency windows and electrode positions extracted are listed in Table 1 and marked in Figure 2 and Supplementary Figures 2, 3. Trial-wise averages of EEG power modulations were z-transformed within each subject and concatenated across subjects. After this process, each EEG time-frequency window and each fMRI voxel contains a time course with one entry for each trial (i.e., trial-wise averages of time-frequency windows for the EEG and beta time-series for the fMRI). We then correlated EEG and fMRI by non-parametric Spearman’s correlation in the temporal dimension in a searchlight manner (Kriegeskorte et al., 2006). For each voxel, we extracted the beta time-series of all neighbors within a sphere of 6 mm radius, which were part of a gray and white matter mask. The resulting beta time-series were then averaged and correlated with the individual time-frequency window of the EEG data. We repeated this approach for each voxel, resulting in an image of correlation coefficients (and *p*-values) in MNI space for each time-frequency window of interest.

To evaluate our approach, we first tested it using the data from the repetitive visual stimulation to locate the 8 Hz time-frequency window of the EEG within the primary visual cortical areas (which therefore were masked). Presented results are FDR corrected for the number of the voxel at a threshold of *p* < 0.05 (one-sided). We used the more general FDR correction instead of FWE correction because the latter is based on random field theory, which might not be applicable to this specific type of non-parametric correlation.

Our primary hypothesis of correlations between trial-wise average power changes in the theta-band time-frequency window and trial-wise BOLD changes in the brainstem was tested using a lower threshold of *p* < 0.005 (one-sided, uncorrected). The latter was chosen because EEG measures only superficial signals, as neuronal activity of the brainstem will be overlaid by cortical activation. Therefore, activity from the brainstem is only represented indirectly and in a minor part of the EEG signal. Furthermore, the nuclei in the brainstem are rather small in comparison to cortical areas. The main source of EEG is derived from the pyramidal cells on the cortex, and the direct contribution from the gray matter in the brainstem is rather small (Olejniczak, 2006). All other trial-to-trial correlations of EEG average power from the selected time-frequency windows and fMRI beta time-series were tested at an FDR-corrected (for the number of the voxel of the gray and white matter mask) threshold of *p* < 0.05 (one-sided).

Furthermore, we included a comparison of correlations between the trigeminal nociception and the control condition. As for the trigeminal nociception, we first calculated correlations between the single-trial beta values of the air puffs and the single-trial EEG responses for the air puffs in the same time-frequency windows as for the nociceptive condition. In a second step, we performed a comparison between the correlation results of the nociception and the air puffs using a permutation approach. For each voxel and time-frequency window, we compared the (Fisher z-transformed) difference of the correlation values of the air puffs and the correlation value of the nociception to a null distribution stemming from the difference of correlation between randomized orders of nociception and control calculated for 50,000 permutations of the randomly selected voxel. As for the main fusion analysis, gamma-band correlation differences were again FDR corrected at an alpha of 0.05, while theta-band correlation differences had to pass a statistical threshold of *p* < 0.005 (uncorrected).

### Correlation With Ratings

In addition, we correlated the subjects’ *z*-scored single-trial intensity ratings of the nociceptive condition with the aforementioned *z*-scored and trial-wise averages of the EEG’s time-frequency windows mentioned in Table 1 using the Pearson correlation coefficient at an alpha level of 0.05 (two-sided).

Similar to our analyses on the fusion of fMRI and EEG, we used *z*-scored trial-wise intensity ratings (instead of the EEG features) to correlate pain intensity and fMRI beta time-series. Here again, we used an FDR-corrected threshold *p* < 0.05.

## Results

### Behavior

The ratings for the painful stimulation of the first trigeminal branch (ammonia) showed significant higher intensity (EEG stand-alone: 53.05 ± 18.12, fMRI stand-alone: 46.26 ± 17.47, EEG-fMRI: 45.69 ± 17.48, with an intensity scale ranging from 0 to 100) and unpleasantness (EEG stand-alone: 7.33 ± 17.17, fMRI stand-alone: 0.95 ± 15.85, EEG-fMRI: 2.02 ± 15.06, with ratings ranging from −50 to 50) ratings than the control condition (air puffs) in each of the three sessions (*p* < 0.01, Wilcoxon signed-rank tests).

### Electroencephalography Features

Extracted EEG features were normally distributed (KS-test) and showed significant differences from 0 for the visual and nociceptive but not for the control condition (air). This applies to both EEG sessions (EEG stand-alone and EEG combined with fMRI), except for the alpha-band after nociception, which became insignificant in the combined session and was therefore dismissed from the fusion analyses between EEG and fMRI. The results are presented in Table 1. The results of the EEG for all 35 subjects in the EEG-only session, the subgroup, which also participated in the EEG-fMRI session, and the EEG-fMRI session are displayed in Supplementary Figure 2.

### Neuroimaging

**FIGURE 3:**
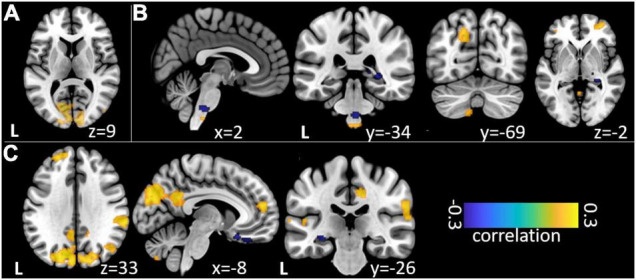
Non-parametric correlations of trial-to-trial variance between EEG event-related band-power changes and fMRI beta time-series. **(A)** Relation of EEG SSVEP and fMRI during repetitive visual stimulation. **(B)** Relation of theta/delta and **(C)** high gamma EEG time-frequency windows during trigeminal nociception.

The repetitive visual stimulation revealed the expected outcome: high activity in primary visual areas and correlations between the SSEP of the EEG and occipital area activation (Figure 3A). Details on the results for the visual stimulation can be found in the Supplementary Section “Results for the Repetitive Visual Stimulation” and Supplementary Figures 2, 3 and Supplementary Tables 2, 3. Similar to the visual stimulation, the trigeminal-nociceptive stimulation also replicated previously published results (Mehnert et al., 2019) on power changes in time-frequency bands of the EEG in the stand-alone session (Figure 2A) as well as during the simultaneous EEG-fMRI measurements (Supplementary Figure 2). Like with visual stimulation, painful stimulation showed an ERS in the frequency band of 3–6 Hz in the parietal-central (Pz) channel ranging from 350 to 1,150 ms after onset. The expected ERD in the alpha frequency range (8–13 Hz) follows. Simultaneously, a wide-ranging synchronization in gamma oscillations occurred, which can be separated into a lower and higher frequency range (Bader, 2019).

In the fMRI stand-alone session, the activation for trigeminal-nociceptive stimulation was similar to previously published results, i.e., FWE-corrected (*p* < 0.05) bilateral activation of pain-related cortical areas (insula, operculum, cerebellum, and somatosensory cortex) and midbrain areas (thalamus) with the dominance of the contralateral hemisphere (Figure 2B and Supplementary Table 2). An activation of the ipsilateral STN, the first relay of the trigeminal nerve in the central nervous system, became significant at a small-volume FWE-corrected (*p* < 0.05) threshold within a sphere of 4 mm around previously published coordinates [MNI coordinates (−6, −39, −45), Stankewitz et al., 2010].

**FIGURE 4:**
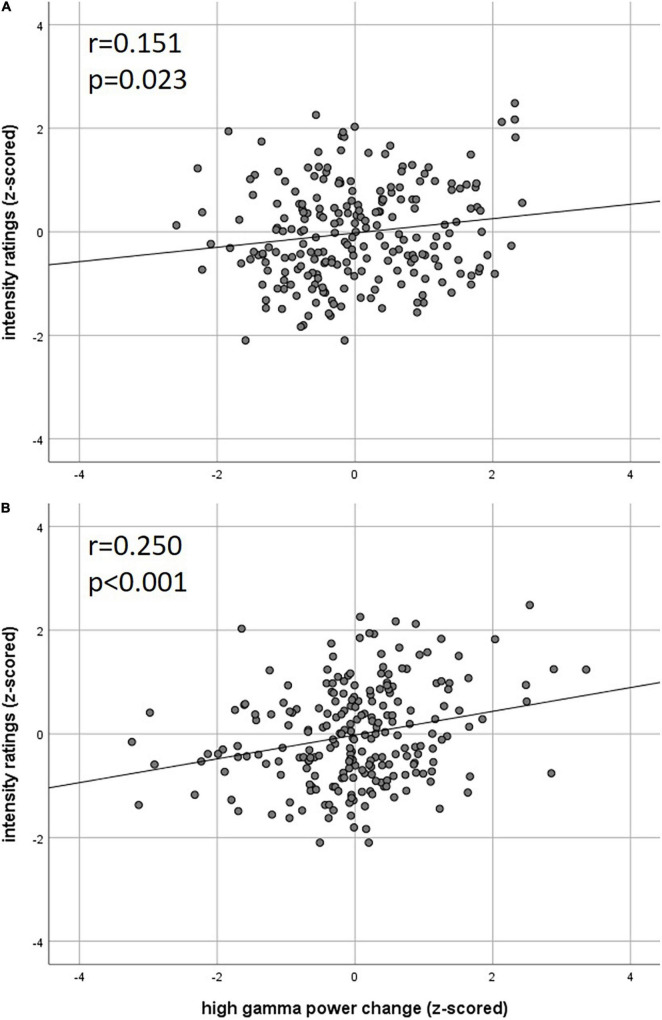
Correlation of intensity ratings with EEG **(A)** theta power and **(B)** high gamma power (*z*-scored rating and power, two-sided Pearson’s correlation).

Our analysis of correlations between EEG power changes in specific time-frequency windows and beta time-series of the fMRI revealed positive relationships between the EEG theta-/delta-band (3–6 Hz) and the bilateral STN (*r* = 0.20), as well as the cerebellum (right: *r* = −0.21; left: *r* = −0.18). The frontal medial gyrus (*r* = 0.23) also showed significant positive correlations between EEG and fMRI. A negative correlation was observed in structures such as the RVM (*r* = −0.21), the entering area of the trigeminal nerve (*r* = −0.22) (Stankewitz et al., 2010), and the entorhinal area (*r* = −0.34). These results are shown in Figure 3B and presented detail in Table 2. These results persist when comparing them to the control condition of air puffs (Supplementary Figure 4 and Supplementary Table 4). Additionally, the strength of these nociceptive stimulus-associated synchronizations was significantly positively correlated with the intensity evaluation of painful stimuli (*r* = 0.151, *p* = 0.023, two-sided test, Figure 4A).

**TABLE 2:** Trial-to-trial correlations between EEG and fMRI for the trigeminal nociception for the theta/delta frequency band at electrode Pz using a statistical threshold of *p* < 0.005 (uncorrected).

Anatomical region (direction of correlation)	Left (ipsilateral)					Right (contralateral)				
	Cluster size	*x*	*y*	*z*	*r*	Cluster size	*x*	*y*	*z*	*r*
Spinal trigeminal nucleus (+)	16	−7	−36	−58	0.198	13	8	−36	−58	0.198
Cerebellum (+)	13	−12	−72	−56	0.182	13	48	−54	−54	0.216
Middle frontal gyrus (+)						156	30	48	4	0.230
Transition zone of sensory trigeminal nerve fibers (−)	30	−14	−18	−30	−0.222					
Rostral ventromedial medulla (−)		–	–	–		53	2	−34	−46	−0.211
Entorhinal area/Parahippocampus (−)		–	–	–		228	20	0	−40	−0.343

Event-related synchronization in the high gamma-band (57–100 Hz) showed positive correlations in bilateral secondary visual association areas (right: *r* = 0.25; left: *r* = 0.29) and in somatosensory areas such as the contralateral SII (*r* = 0.27), middle cingulate cortex (*r* = 0.25), and insula (*r* = 0.23). In addition, negative correlation was found bilaterally in the primary visual cortex (right: *r* = −0.24; left: *r* = −0.20) and in the ipsilateral insula (*r* = −0.23). These results are presented in Figure 3C and Table 3. No significant correlation between fMRI beta time-series and EEG power changes in the alpha and low gamma range were observed at the chosen statistical threshold. These results persist when comparing them to the control condition of air puffs (Supplementary Figure 4 and Supplementary Table 4). We further found a significantly positive correlation between the gamma-band and the individual intensity ratings (*r* = 0.25, *p* < 0.001, two-sided test, Figure 4B). Correlations of intensity ratings and fMRI beta time-series were not significant at an FDR-corrected threshold of *p* < 0.05.

**TABLE 3:** Trial-to-trial correlations between EEG and fMRI for the trigeminal nociception for the high gamma frequency band at electrode Pz using a statistical threshold of *p* < 0.05 (FDR corrected for the considered number of the voxel).

Anatomical region (direction of correlation)	Left (ipsilateral)					Right (contralateral)				
	Cluster size	x	y	z	r	Cluster size	x	y	z	r
Visual cortex (−)	22	−22	−100	−12	−0.204	148	16	−100	−12	−0.247
Insula (−)	35	−42	0	20	−0.234					
Orbital gyri (−)	235	−10	18	−24	−0.264	58	10	28	−26	−0.237
Middle temporal gyrus (+)	1907	−56	−64	14	0.299	295	52	−46	8	0.253
Insula (+)						29	36	−6	12	0.225
Middle cingulate cortex (+)						78	14	−22	36	0.246
SII (+)						356	66	−34	30	0.265
Middle occipital gyrus (+)						197	46	−68	26	0.205
Cuneus/Precuneus (+)	636	−16	−76	28	0.258	734	18	−76	28	0.217

## Discussion

The simultaneous recording of EEG and fMRI has received increased attention in basic research and clinical translation (Debener et al., 2006; Ullsperger and Debener, 2010; Huster et al., 2012) since it has the potency to overcome a fundamental problem in neuroimaging: the imbalance between the temporal and spatial resolution of electrophysiological and hemodynamic responses. This study serves as a bridge to translate our well-studied experimental paradigm on trigeminal nociception induced by gaseous ammonia (Stankewitz et al., 2010) to non-invasive simultaneous EEG-fMRI to gain further insights into trigeminal pain processing in humans. For this purpose, the primary goal of this study is to validate and evaluate this approach together with a novel way of fusing EEG and fMRI data.

As a first step, we reevaluated our paradigm in two stand-alone sessions of separate EEG and fMRI measurements. For both modalities, we were able to reproduce previously published results for trigeminal nociception and also for repetitive visual stimulation. We used the latter to validate our analytical approach by fusing data from both modalities during the simultaneous experimental session. Regarding trigeminal nociceptive stimuli, we observed in fMRI, in both stand-alone and simultaneous sessions, activations in areas such as the thalamus, primary and secondary somatosensory cortices, insula, and cingulate cortex. These results (Supplementary Table 2) are in line with previous studies dealing with the central processing of painful, in particular, trigeminal nociceptive stimuli (Peyron et al., 2000; Treede et al., 2000; Apkarian et al., 2005; Tracey and Mantyh, 2007; Stankewitz et al., 2010). The results of the time-frequency analyses in the EEG of the EEG stand-alone session reproduced previously published findings (Bader, 2019; Mehnert et al., 2019) despite a smaller cohort size (Table 1). The EEG results from the stand-alone session were further used as a reference to confirm the reliability of the extensive cleaning of artifacts caused by influences of EEG signals on MRI during the simultaneous collection of both modalities. In the combined EEG-fMRI session, we observed a slight reduction in the induced power, although the previously observed stimulus-associated effects were rather robust in explicit time-frequency bands (Supplementary Figure 2).

### Non-parametric Correlation of Intertrial Variation in Electroencephalography and Functional Magnetic Resonance Imaging

In addition to the reevaluation of our results for simultaneous EEG-fMRI, we carefully validated our analytical approach for fusing both modalities with the repetitive visual stimulation, as here the hypothesis is clear: a strong correlation between trial-to-trial variations in the fMRI of the primary visual cortex and the SSEP in the EEG. Several approaches to fuse EEG and fMRI exist (Mulert and Lemieux, 2010; Abreu et al., 2018), and they all have their advantages and disadvantages. Early EEG-fMRI studies aimed at correlating the raw (but temporally shifted or convolved by a hemodynamic response function) time courses of both modalities (Moosmann et al., 2003; Ritter et al., 2009), presuming a quasi-linear relationship and artifact-free data. Later approaches mostly used ERP components, which are less stable than the power changes of time-frequency bands (Varela et al., 2001). As a result, they need a high number of repetitions, which are subsequently used as an additional regressor for the fMRI analysis, mostly as a parametric modulator, which also assumes linearity (Andreou et al., 2017). Notably, EEG and fMRI measure different signals of activation, and an assumption of linearity might be misleading to research their relationship. To overcome this issue, there are multiple ways to use multivariate approaches (Dähne et al., 2015), which fuse multimodal data with the disadvantage of the need for a high number of repetitions for adequate cross-validation (Dähne et al., 2013) or a high number of conditions exploiting the function of interest (Cichy et al., 2016). Thus, these approaches seem only possible for higher cognitive functions.

As the number of repetitions and the number of conditions to deliver trigeminal pain are limited, we decided to examine non-linear rank correlations of trial-to-trial variations of both modalities in an EEG-informed fMRI fashion, where the z-transformed single-trial responses of the individual participants were concatenated. To achieve this, the stimulation-associated responses in the EEG of previously published time-frequency windows were correlated with estimates of the hemodynamic responses of the individual stimuli from the fMRI. Our approach has the advantage that relatively few trials are necessary, and it is easily extendable to a multivariate fashion, e.g., by using a support vector regression (Bogler et al., 2014) during the searchlight approach (Allefeld and Haynes, 2014).

### Trigeminal Nociception in Electroencephalography-Informed Functional Magnetic Resonance Imaging

Our main finding during trigeminal nociception is a significant relationship between the theta/delta frequency bands, which corresponds to the time-frequency equivalent of a nociceptive evoked potential (Makeig et al., 2004) and is also correlated to the individually perceived intensity of the painful stimulation and the STN as well as the RVM. As hypothesized, this time-frequency window shows a significant correlation with the corresponding activation estimates in certain brainstem regions (see Figure 3B and Table 2) including the STN, the first relay station of the trigeminal peripheral nerve (Borsook et al., 2003; Stankewitz et al., 2010; Schulte et al., 2016). The result is that the variability of the EEG signal is more covariant with STN activation than with other somatosensory discriminating regions of the pain matrix, such as SI or SII, which could lead to the conclusion that the ascending pain signal is processed independently of the somatotopic assignment in SI (Petrovic et al., 2004). The negative relation with the RVM, which is part of the descending antinociceptive system (Heinricher et al., 1989; Ossipov et al., 2010), contrasts this positive relation. This negative association might reflect the results of the animal study by Heinricher et al. (1989): they showed that the spontaneous activity of “on” and “off” cells in the RVM is modulated by painful stimuli at a very early stage of central pain processing. An opposite correlation of the nociceptive signal from the STN to the RVM with decreasing strength of the EP suggests that, in addition to nociception, antinociceptive modulation is initiated simultaneously to (pro)nociceptive ascending pain processing. In conclusion, the strength of the pain-induced early evoked synchronizations in the theta/delta frequency band, which is the time-frequency equivalent of the nociceptive evoked potential, indicates an early trial-to-trial modulation of trigeminal nociceptive stimuli already at the brainstem level.

In the gamma frequency range, we observed a correlation between EEG and fMRI in numerous cortical structures. In addition to positive correlations with regions generally associated with the pain matrix, such as the contralateral insula, middle cingulate cortex, and SII, extra-sensory cortical structures such as visual (cuneus/precuneus) and visual-associative areas (temporal gyrus, occipital medial gyrus) also showed a positive correlation between the gamma power modulation in EEG and the single-trial activation in fMRI (Figure 3C and Table 3). This can be explained by the full range of neuronal structures for processing extrinsic stimuli that are inevitably obtained by extending the time window of observation (Senkowski et al., 2014). Furthermore, it has been shown that fMRI results and gamma power changes after nociceptive input are not specific to pain and can also be induced by other attentive paradigms (Legrain et al., 2011) and hence reflect a more general salience system, which can also explain the correlation to visual and frontal areas. However, the individual variability in the strength of nociceptive-evoked gamma oscillations suggests that central sensory processing occurs at both motivational-affective and purely sensory discriminatory levels. Consequently, power changes in this time-frequency window are significantly positively correlated with individuals’ intensity ratings (Figure 4B), echoing the previous findings (Gross et al., 2007; Hauck et al., 2007; Schulz et al., 2012; Zhang et al., 2012; Tu et al., 2016). This is corroborated by the positive correlations of this gamma oscillation with BOLD signals in areas of the lateral as well as the medial pain pathway. Interestingly, the contralateral and ipsilateral insula correlate in opposite directions with the EEG’s power changes. This might be explained by feedback transmission, which could be dependent on the stimulated side. In conclusion, the gamma frequency range might not exclusively indicate the processing of nociceptive-sensory stimuli but reveals associations to a wide range of structures, suggesting both motivational-affective and sensory-discriminative processing recruiting associative areas (secondary visual cortex, medial occipital gyrus, temporal gyrus), while primary structures (primary visual cortex) show an opposite direction of influence on both signals.

### Conclusion

Our study validates an experimental trigeminal nociceptive paradigm for simultaneous EEG-fMRI and a novel approach for EEG-informed fMRI analysis. While our findings on the experimental side should be extended to clinical cohorts such as migraine or cluster headache patients, our analytical approach may be adapted to any multimodal data analysis and possibly extended to a multivariate approach.

## Data Availability Statement

The raw data supporting the conclusions of this article will be made available by the authors, to the researchers meeting the criteria to access the confidential data.

## Ethics Statement

The studies involving human participants were reviewed and approved by the Ärztekammer Hamburg, PV 4896. The patients/participants provided their written informed consent to participate in this study.

## Author Contributions

JM and AM designed the experiment. HB and K-PP collected the data. JM and HB analyzed the data. All authors wrote and reviewed the final manuscript.

## Conflict of Interest

The study was funded by the Deutsche Forschungsgemeinschaft (DFG, German Research Foundation) – SFB936-178316478 – A5 (AM). The funder was not involved in the study design, collection, analysis, interpretation of data, the writing of this article or the decision to submit it for publication. The remaining authors declare that the research was conducted in the absence of any commercial or financial relationships that could be construed as a potential conflict of interest.

## Publisher’s Note

All claims expressed in this article are solely those of the authors and do not necessarily represent those of their affiliated organizations, or those of the publisher, the editors and the reviewers. Any product that may be evaluated in this article, or claim that may be made by its manufacturer, is not guaranteed or endorsed by the publisher.

## Footnotes

1
https://www.fieldtriptoolbox.org/

2
http://www.fil.ion.ucl.ac.uk/spm/
