# Supplementary material for: High-Density Electroencephalography-Informed Multiband Functional Magnetic Resonance Imaging Reveals Rhythm-Specific Activations Within the Trigeminal Nociceptive Network
Source: Front Neurosci. 2022 May 16;16:802239. doi: 10.3389/fnins.2022.802239 (PMC9149083; doi:10.3389/fnins.2022.802239)
Supplement: Supplementary file 1 [file Data_Sheet_1.docx]

**Supplement**

***Experimental Design***


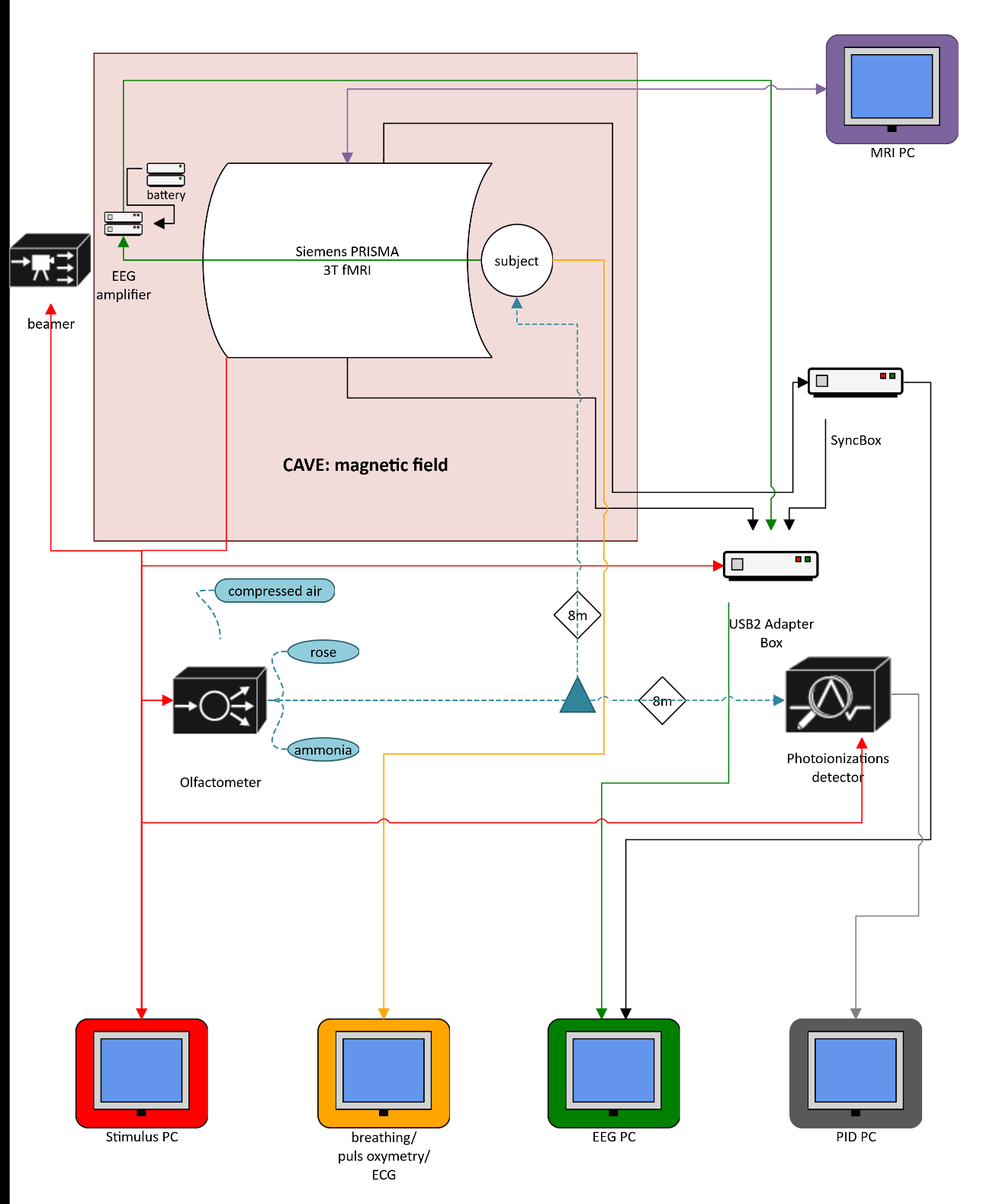
*Supplementary Figure SF1: Sketch of the setup for the simultaneous EEG-fMRI acquisition*.

***EEG***


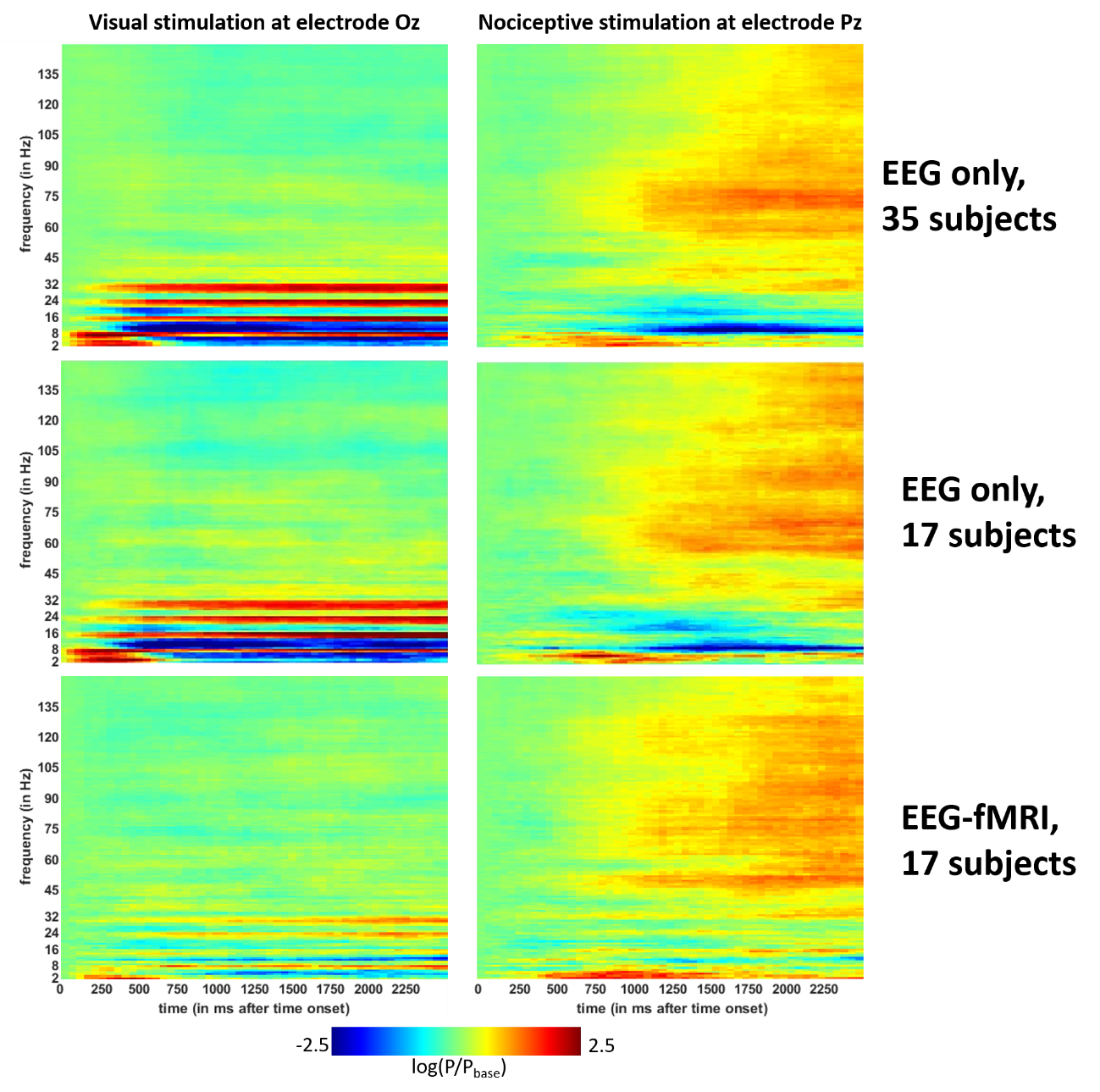


*Supplementary Figure SF2: Results for the EEG for the visual condition (left) and trigeminal nociception (right) for three groups: subjects participating in the EEG-only session, the subgroup, which also participated in the EEG-fMRI, and the results for the EEG-fMRI session.*

***Results for the Repetitive Visual Stimulation***

The repetitive visual condition consisting of an 8 Hz flickering checkerboard stimulation replicated the expected results in the EEG standalone session (Supplementary Figure SF3A) as well as in the simultaneous EEG-fMRI session (Supplementary Figure 2, left hand side), namely a SSEP [47] at the flickering frequency, in line with our previous publication [37]. In detail, the group average of time-frequency analyses revealed an early ERS in the frequency band of 2 – 9 Hz at the occipito-central (Oz) electrode in the time range of 100 to 500 ms, which is the time-frequency equivalent of the event-related response (ERD) but not subject of our statistical analyses. This was followed by an ERD in this frequency range (indicated as blue color). The SSVEP and its higher (sub-) harmonics at 16, 24 and 32 Hz showed a stable effect across the whole considered temporal window. An expected ERD in the alpha-band arose from about 350 ms after stimulus onset. These results reproduced the expectations for SSEP stimulations and specifically our previous publication on this experimental protocol [37].

Also, the standalone fMRI of the visual condition replicated previously published results [e.g. 60], i.e. FWE-corrected (p<0.05) activations in primary visual cortex as well as bilaterally in the lateral geniculate nuclei (LGN), which we show in Supplementary Figure 3B (and more detailed in Supplementary Table ST 1).

As expected, the trial-to-trial analysis of the simultaneous EEG-fMRI session revealed significant FDR-corrected (p<0.05) correlations of the SSVEP (in the time frequency window of 8 Hz and between 100 and 2000 ms after stimulus onset) with primary visual cortex (r=0.21, p<0.001) as well as in several other secondary and associative areas (Figure 2A, Supplementary Table ST2). This verifies the validity of the preprocessing as well as the chosen analytical method.


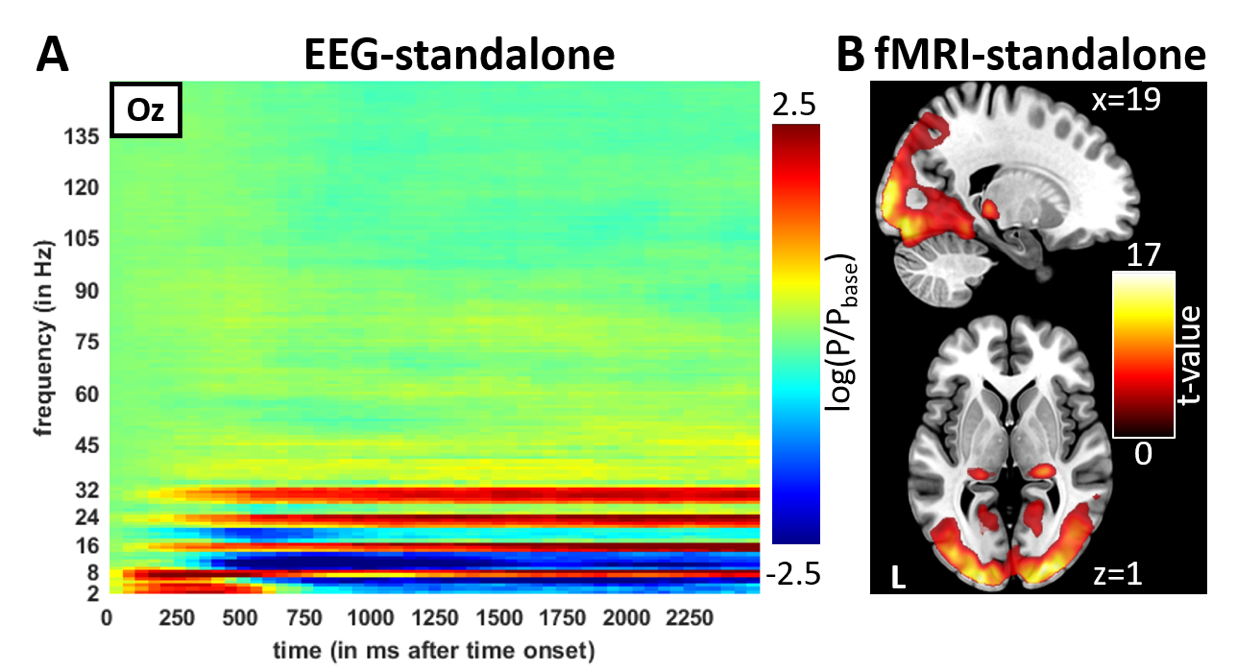


*Supplementary Figure SF3: Result from the standalone measurements during repetitive visual stimulation in EEG and fMRI. A) Averages of stimulus induces power changes in time-frequency bands of the visual condition in the EEG and B) activity seen by the fMRI (p<0.001, uncorrected). The time-frequency window of interest are framed in white.*

*Supplementary Table ST1: FMRI activation for the repetitive visual stimulation (standalone) at an FWE-corrected statistical threshold of p<0.05 and a minimal cluster extent of 30 voxel.*

| **Anatomical region** | **Cluster extent** | **MNI coordinate** | | | **t-value** |
| --- | --- | --- | --- | --- | --- |
|  | **[voxel]** | **x** | **y** | **z** |  |
| **Occipital lobe/ visual cortex** | 84062 | 15 | -89 | -10 | 17.40 |
| **r Thalamus** | 780 | 24 | -27 | -3 | 10.64 |
| **l Thalamus** | 511 | -19 | -31 | -2 | 10.16 |
| **l Cerebellum** | 50 | -9 | -77 | -39 | 6.66 |
| **l Inferior parietal lobule** | 72 | -27 | -51 | 47 | 6.56 |
| **l Superior parietal lobule** | 31 | -23 | -70 | 42 | 6.42 |
| **r Superior parietal lobule** | 35 | 26 | -52 | 47 | 6.18 |
| l = left, r = right | | | | | |

*Table ST2: Trial-to-trial correlations between EEG and fMRI for the repetitive visual stimulation in the time-frequency window of the flicker (i.e. the SSVEP) at electrode Oz using a statistical threshold of p<0.05 (FDR-corrected for the considered number of voxel).*

| **Anatomical region** | **Cluster size** | **MNI coordinates** | | | **Correlation** |
| --- | --- | --- | --- | --- | --- |
|  | **[voxel]** | **x** | **y** | **z** | **[Spearman‘s r]** |
| **l Lingula g** | 2763 | -16 | -74 | -5 | 0.250 |
| **l Lingula g** | 79 | -18 | -51 | -3 | 0.217 |
| **l Fusiform g** | 54 | -27 | -50 | -13 | 0.197 |
| **r Calcarine cortex** | 700 | 13 | -92 | 6 | 0.205 |
| **r Middle occipital g** | 73 | 48 | -75 | 5 | 0.191 |
| **l Cuneus** | 61 | -4 | -82 | 26 | 0.173 |
| **r Lingula g** | 192 | 8 | -70 | 0 | 0.169 |
| l = left, r = right, g = gyrus | | | | | |

***Results for the Trigeminal Nociceptive Stimulation***

*Supplementary Table ST3: FMRI activation for the trigeminal nociception (standalone) at an FWE-corrected statistical threshold of p<0.05 and a minimal cluster extent of 30 voxel.*

| **Anatomical region** | **Cluster extent** | **MNI coordinate** | | | **t-value** |  |
| --- | --- | --- | --- | --- | --- | --- |
|  | **[voxel]** | **x** | **y** | **z** |  |  |
| r Insula | 10162 | 35 | 23 | 3 | 10.78 | |
| r Postcentral g | 3172 | 40 | -15 | 39 | 10.70 | |
| r Supramarginal g | 4682 | 61 | -25 | 24 | 10.27 | |
| l Cerebellum | 1268 | -12 | -75 | -35 | 9.95 | |
| l Cerebellum | 1285 | -17 | -66 | -23 | 9.48 | |
| r Midcingulate cortex | 3059 | 5 | 19 | 31 | 9.37 | |
| l Postcentral g | 748 | -38 | -18 | 41 | 9.01 | |
| r Supplementary motor area | 393 | 13 | 4 | 70 | 8.82 | |
| r Middle frontal g | 490 | 48 | 48 | 10 | 8.72 | |
| l Insula | 222 | -39 | -5 | -6 | 8.45 | |
| r Thalamus | 384 | 8 | -18 | -3 | 8.16 | |
| l Cerebellum | 619 | -37 | -57 | -52 | 7.96 | |
| Periaqueductal grey | 50 | 10 | -15 | -10 | 7.91 | |
| r Middle Frontal g | 387 | 32 | 48 | 28 | 7.71 | |
| l Lingula g | 43 | -21 | -54 | -3 | 7.61 | |
| r Precentral g | 91 | 45 | 6 | 32 | 7.54 | |
| l Cerebellum | 38 | -30 | -56 | -35 | 7.41 | |
| l Cuneus | 74 | -7 | -89 | 27 | 7.41 | |
| l Cerebellum | 87 | -25 | -76 | -44 | 7.33 | |
| r Middle Frontal g | 460 | 41 | 44 | 25 | 7.23 | |
| r Cuneus | 299 | 14 | -65 | 35 | 7.18 | |
| r Cerebellum | 313 | 20 | -62 | -25 | 7.16 | |
| l Postcentral g | 69 | -44 | -11 | 51 | 7.06 | |
| l Postcentral g | 65 | -65 | -20 | 24 | 7.06 | |
| r Middle Frontal g | 81 | 46 | 42 | 2 | 6.99 | |
| l Superior occipital g | 122 | -12 | -79 | 38 | 6.98 | |
| l Inferior parietal g | 45 | -54 | -34 | 43 | 6.89 | |
| l Supplementary motor area | 39 | -4 | -5 | 61 | 6.79 | |
| Calcarine cortex | 34 | -3 | -72 | 16 | 6.71 | |
| l = left, r = right, g = gyrus | | | | | |  |

***Results for the Comparison of Trigeminal Nociception and Control Condition***


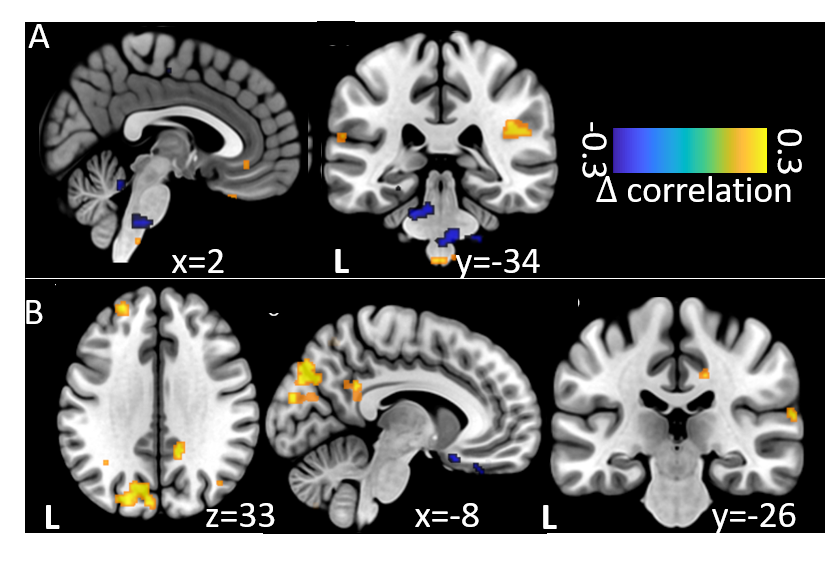


*Supplementary Figure SF4: Differences between the correlation of the control condition and the trigeminal nociception for (A) theta/delta band EEG and the fMRI beta-time series at an statistical threshold of p<0.005 (uncorrected), and for (B) gamma band EEG and the fMRI beta-time series at an statistical threshold of p<0.05 (FDR-corrected),*

*Supplementary Table ST4: Differences between the correlation of the control condition and the trigeminal nociception for theta/delta band EEG and the fMRI beta-time series at an statistical threshold of p<0.005 (uncorrected), and for gamma band EEG and the fMRI beta-time series at an statistical threshold of p<0.005 (FDR-corrected),*

| **Anatomical region** | **Cluster size** | **MNI coordinates** | | | **Difference in correlation** |
| --- | --- | --- | --- | --- | --- |
|  | **[voxel]** | **x** | **y** | **z** | **[∆ Spearman‘s r]** |
| **gamma band** |  |  |  |  |  |
| L Cerebellum | 13 | -22 | -54 | -52 | 0.318 |
| L Subcallosal Area | 65 | -6 | 14 | -20 | -0.281 |
| R Medial Orbital G | 11 | 16 | 54 | -18 | -0.283 |
| L Angular G | 294 | -44 | -68 | 20 | 0.392 |
| R Posterior Insula | 39 | 34 | -8 | 6 | 0.321 |
| R Thalamus | 18 | 4 | -10 | 10 | 0.337 |
| R Precuneus | 44 | 22 | -66 | 22 | 0.323 |
| L Cuneus | 428 | -20 | -80 | 28 | 0.373 |
| R Middle Occipital G | 123 | 44 | -72 | 22 | 0.353 |
| R Cuneus | 58 | 6 | -74 | 22 | 0.347 |
| L Precuneus | 67 | -2 | -54 | 30 | 0.347 |
| R Posterior Cingulate G | 60 | 16 | -48 | 32 | 0.401 |
| R Supramarginal G | 11 | 60 | -44 | 28 | 0.303 |
| L Superior Frontal G | 25 | -26 | 50 | 32 | 0.329 |
| L Superior Parietal Lobule | 21 | -14 | -52 | 64 | 0.356 |
| **theta/delta band** |  |  |  |  |  |
| R Cerebellum | 14 | 24 | -58 | -56 | 0.250 |
| L Cerebellum | 11 | -12 | -42 | -56 | 0.248 |
| R Cerebellum | 13 | 10 | -72 | -54 | 0.236 |
| R STN | 10 | 10 | -40 | -56 | 0.248 |
| R Cerebellum | 20 | 12 | -82 | -48 | 0.244 |
| L Cerebellum | 16 | -8 | -80 | -50 | 0.250 |
| R Cerebellum | 126 | 24 | -38 | -44 | -0.214 |
| R Fusiform G | 68 | 38 | -14 | -42 | -0.214 |
| L Temporal Pole | 50 | -32 | 6 | -42 | -0.216 |
| L Fusiform G | 19 | -38 | -10 | -38 | -0.214 |
| R Temporal Pole | 135 | 22 | 2 | -46 | -0.214 |
| R Cerebellum WM | 12 | 12 | -28 | -40 | -0.217 |
| R Fusiform G | 53 | 36 | -18 | -28 | -0.216 |
| L Cerebellum WM | 84 | -4 | -44 | -24 | -0.213 |
| Transitional Zone of sensory trigeminal nerve fibers | 20 | -10 | -18 | -32 | -0.214 |
| L Fusiform G | 41 | -36 | -44 | -26 | -0.214 |
| R Inferior Temporal G | 11 | 54 | -62 | -22 | -0.214 |
| Cerebellar Vermal Lobules I-V | 12 | 0 | -42 | -20 | -0.215 |
| L Subcallosal Area | 26 | -10 | 14 | -16 | -0.214 |
| L Inferior Occipital G | 15 | -42 | -82 | -10 | -0.213 |
| R Anterior Insula | 36 | 30 | 8 | -14 | -0.214 |
| L Inferior Temporal G | 95 | -50 | -52 | -8 | 0.296 |
| L Posterior Insula | 12 | -34 | -20 | 10 | -0.215 |
| L Anterior Insula | 12 | -30 | 12 | 14 | 0.254 |
| R Planum Temporale | 58 | 44 | -36 | 22 | 0.293 |
| L Superior Occipital G | 22 | -16 | -86 | 32 | 0.241 |
| L Precuneus | 55 | -16 | -70 | 34 | 0.294 |
| L Middle Cingulate G | 31 | -4 | -18 | 40 | 0.267 |
| R Supplementary Motor Cortex | 26 | 4 | -14 | 52 | -0.216 |
| L Superior Parietal Lobule | 23 | -24 | -60 | 64 | 0.303 |
| R Cerebellum | 14 | 24 | -58 | -56 | 0.250 |
| l = left, r = right, g = gyrus | | | | | |
